# The impact of environmental factors on respiratory tract microbiome and respiratory system diseases

**DOI:** 10.1186/s40001-025-02517-3

**Published:** 2025-04-04

**Authors:** Yutao Ge, Guo Tang, Yawen Fu, Peng Deng, Rong Yao

**Affiliations:** Emergency Department of West China Hospital, Sichuan University, No. 37 Guoxue Alley, Chengdu, 610041 Sichuan China

**Keywords:** Respiratory microbiome, Temperature, Humidity, Air pollutants, Gut–lung axis

## Abstract

The respiratory tract microbiome, a complex ecosystem of microorganisms colonizing the respiratory mucous layers and epithelial surfaces along with their associated microenvironment, plays a vital role in maintaining respiratory function and promoting the maturation of the respiratory immune system. Current research suggests that environmental changes can disrupt the respiratory microbiota, potentially leading to disease. This review summarizes existing research on the impact of environmental factors on the respiratory microbiome and associated diseases, aiming to offer new insights into the prevention and treatment of respiratory disease.

## Introduction

The respiratory microbiome is a multi-kingdom microbial ecosystem inhabiting the respiratory tract mucosal layer and epithelial surfaces, from the nasal cavity to the alveoli, along with the microenvironment on which these microorganisms depend [1]. The normal respiratory microbiota serves as a barrier against pathogenic microorganisms entering the lungs, and is essential for maintaining the health of the respiratory system [2, 3]. When the respiratory microbiome becomes imbalanced and microbial diversity decreases, it can trigger or exacerbate respiratory diseases [4]. Due to the direct connection of the respiratory tract to the external environment, the respiratory microbiota is exposed to various environmental factors. Although some environmental factors have been found to potentially affect the stability and diversity of the respiratory microbiota, comprehensive systematic research remains limited. This review aims to summarize current research on the impact of environmental factors on the respiratory microbiome and related diseases, with the goal of providing new insights for the prevention and treatment of respiratory diseases.

The literature search for this study was conducted in the following databases: PubMed, Web of Science, and Google Scholar. The search period spanned from January 2010 to February 2025, with the search terms including:

**Table:** 

Respiratory tract	(lung[MeSH Terms]) OR (lungs[Title/Abstract]) OR (airway[Title/Abstract]) OR (pulmonary[Title/Abstract]) OR (alveolar[Title/Abstract]) OR (nasopharynx[Title/Abstract]) OR (nasopharyngeal[Title/Abstract])
Microbiome	(Microbio* [Title/Abstract])
Temperature	(temperature[MeSH Terms]) OR (thermal exposure[Title/Abstract]) OR (ambient temperature[Title/Abstract]) OR (body temperature[Title/Abstract])
Heat	(heat[Title/Abstract]) OR (hyperthermia[Title/Abstract]) OR (high temperature[Title/Abstract]) OR (thermal stress[Title/Abstract]) OR (heat exposure[Title/Abstract]) OR (heat wave[Title/Abstract]) OR (extreme heat[Title/Abstract]) OR (environmental hyperthermia[Title/Abstract]) OR (exertional heat[Title/Abstract]) OR (heatstroke[Title/Abstract]) OR (heat stroke[Title/Abstract]) OR (heat shock[Title/Abstract]) OR (heat exhaustion[Title/Abstract]) OR (heat-related illness[Title/Abstract]) OR (heat syncope[Title/Abstract]) OR (heat cramps[Title/Abstract])
Humidity	(humidity[Title/Abstract]) OR (moisture[Title/Abstract]) OR (humid climate[Title/Abstract])
Environment	(environment[Title/Abstract]) OR (environmental[Title/Abstract]) OR (climate[Title/Abstract]) OR (season[Title/Abstract]) OR (seasonality[Title/Abstract])
Air pollution	(air pollution[Title/Abstract]) OR (PM2.5[Title/Abstract]) OR (PM10[Title/Abstract]) OR (ozone[Title/Abstract])
Altitude	altitude*[Title/Abstract]
Lung injury	("Lung Injury"[Mesh]) OR (Lung Injuries[Title/Abstract]) OR (Pulmonary Injury[Title/Abstract])
COPD	Chronic Obstructive Pulmonary Disease[Title/Abstract]
Asthma	Asthma [Title/Abstract]
Lung Cancer	Lung Cancer [Title/Abstract]
Interstitial lung disease	Interstitial lung disease [Title/Abstract]

The criteria for literature screening were: (i) the research was original (such as clinical trials, cohort studies, and case–control studies); (ii) it addressed the influence of the microbiota on lung health or diseases; (iii) the articles appeared in peer-reviewed journals. We excluded case reports and studies of lower quality (such as those with insufficient sample sizes or inadequate methodologies). All the literature was screened and quality-assessed by two independent researchers, with a total of 94 studies ultimately included. We qualitatively analyzed these studies, focusing on discussing their similarities and differences, and proposed the potential role of the microbiome in pulmonary health.

## The composition of the respiratory microbiome

The respiratory system is crucial for gas exchange between the human body and the external environment. Historically, the lower respiratory tract was considered sterile. However, advances in genetic technologies and the widespread use of high-throughput sequencing have gradually revealed the presence of microbial communities in the lower respiratory tract and lungs. Colonization of the respiratory microbiota begins shortly after birth, with bacterial DNA detectable in tracheal aspirates from newborns within 24 h [5].

The respiratory tract is divided into the upper and lower sections, with the cricoid cartilage serving as the boundary. The upper respiratory tract includes the nasal cavity, paranasal sinuses, pharynx, and the supraglottic portion of the larynx, while the lower respiratory tract comprises the trachea, bronchi, bronchioles, respiratory bronchioles, alveolar ducts, and alveoli. The upper respiratory tract is continuously exposed to the external environment, lacking physical barriers, and its microbial composition directly impacts respiratory health. The microbiota of the oropharynx and nasopharynx share similarities, particularly at birth, when both are predominantly colonized by *Streptococcus* [6]. By one week postpartum, the nasopharynx is predominantly colonized by *Moraxella* and *Haemophilus*. By one month, the nasopharyngeal microbiome exhibits a high abundance of *Moraxella*, *Staphylococcus*, *Streptococcus*, *Corynebacterium*, and *Haemophilus* [7, 8]. However, the oropharyngeal microbiota tends to remain relatively stable over time, predominantly consisting of *Streptococcus* [6]. Additionally, the oropharyngeal microbiome also comprises *Neisseria*, *Prevotella*, *Veillonella*, and *Haemophilus* [7–9].

Current research indicates that the microbiota of the upper and lower respiratory tracts exhibits a high degree of homology. The microbiota of the lower respiratory tract primarily originates from the oropharynx, migrating to the lower respiratory tract via aspiration. However, the lower respiratory tract microbiota possesses distinct characteristics [10, 11]. The lung microbiome is predominantly composed of *Proteus*, *Prevotella*, *Streptococcus*, and *Veillonella* [12–14].

## Relationship between respiratory microbiome alterations and respiratory diseases

The stability of the respiratory microbiome is crucial for maintaining normal respiratory function and facilitating the maturation of the neonatal immune system [5]. Normal respiratory microbiota aids in pathogen clearance, regulates host immune cell functions [15], and prevents pathogen colonization through competitive exclusion or the production of inhibitory substances [13]. An imbalance in the normal composition of the respiratory microbiota can lead to immune dysfunction, potentially triggering or exacerbating respiratory diseases [16–20]. Recent research has confirmed substantial differences in the lung microbiome between individuals with prevalent respiratory diseases and healthy controls, aberrant microbial populations (dysbiosis) have been confirmed in many prevalent lung diseases that are traditionally not considered to be of microbial origin [2, 21].

### Chronic obstructive pulmonary disease (COPD)

Compared with the healthy control group, stable COPD patients showed a decrease in pulmonary microbiota diversity, characterized by an increased presence of *Moraxella*, *Streptococcus*, *Veillonella* and *Prevotella* [22–24], while those experiencing acute exacerbations of COPD exhibited elevated levels of *Pseudomonas aeruginosa* and *Klebsiella pneumoniae* in their lung microbiome [25, 26].

A prospective study of 101 patients with the exacerbation of COPD revealed that there was an increased abundance of *Haemophilus*, whereas the abundances of *Prevotella* and *Veillonella* decreased [27]. A cross-sectional study of 72 COPD patients revealed that airflow obstruction correlated with an elevated relative abundance of *Pseudomonas* in the sputum [28].

A study investigating the association between COPD severity and changes in the microbiome revealed that as the disease advanced and ventilatory function progressively worsened, the microbial diversity in the sputum of advanced COPD patients diminished, with the main microbial phyla comprising *Proteobacteria*, *Firmicutes*, *Actinobacteria*, and *Bacteroidota* [29].

A multivariate modeling analysis of sputum microbiome, host transcriptome, and proteome data demonstrated that in the host-microbiome interplay within the airways of COPD patients, *Moraxella* and *Haemophilus* were predominantly involved in linking host interferon and pro-inflammatory signaling pathways with neutrophil inflammation [23].

Distinct respiratory tract microbiota have also been confirmed among various inflammatory endotypes of COPD. A study that analyzed sputum samples from 510 subjects revealed that the respiratory tract microbiome in neutrophilic COPD was heterogeneous. One subgroup is characterized by a predominance of *Haemophilus*. Another subgroup represents a neutrophilic subgroup characterized by a balanced microbial composition [30]. A cohort study involving 87 COPD patients demonstrated an association between neutrophilic inflammation and elevated *Moraxella* levels in sputum samples [31]. Analysis of a 43-subject cohort identified positive correlations between neutrophilic predominance and increased sputum abundances of *Haemophilus* and *Neisseria* [23]. Neutrophilic inflammation exhibited lower microbial diversity compared to other COPD inflammatory endotypes [32]. Conversely, eosinophilic inflammation in COPD correlated with reduced bacterial infections and enhanced lung microbiota diversity [28, 30–34]. Therefore, inflammation-specific alterations in respiratory microbiota composition may serve as viable biomarkers to track and anticipate inflammatory phenotypes in COPD patients.

Nonetheless, a study examining whether the lower respiratory tract microbiome composition could forecast the risk of future exacerbations in COPD patients found that the relative abundance of various microbial communities displayed considerable variability among samples and individuals, and that the individual differences in the lower respiratory tract microbiome were far greater than the differences between frequent and infrequent exacerbators [35]. Consequently, substantial experimental validation is required to determine if the respiratory tract microbiome can be utilized as a biomarker for predicting disease severity and inflammatory states in COPD.

### Asthma

Asthma patients demonstrate elevated abundances of *Proteus*, *Haemophilus*, *Moraxella*, and *Neisseria* in their pulmonary microbiome compared to healthy controls [21, 36–39]. Sputum samples from asthma patients show a higher relative abundance of *Neisseria* and *Proteus* and increased microbial diversity, whereas *Firmicutes* and *Actinobacteria* dominate in healthy individuals [40]. *Actinobacteria* abundance is significantly higher in severe asthma patients relative to healthy controls or individuals with mild-to-moderate asthma [41].

Asthmatic children exhibit elevated nasal abundances of *Moraxella* and *Haemophilus* compared to healthy controls [42, 43]. A cohort study involving 234 Australian children (2- to 12-month-old) with asthma revealed that their nasopharyngeal microbiome was dominated by *Haemophilus*, *Streptococcus*, *Moraxella*, *Alloiococcus*, *Corynebacterium*, and *Staphylococcus* [44].

Asthma and COPD demonstrate overlapping inflammatory phenotypes (neutrophilic and eosinophilic infiltration) with analogous microbiome profiles. Patients presenting with neutrophilic asthma show reduced microbial diversity in the respiratory microbiome relative to other inflammatory endotypes [31, 38, 45, 46].

In contrast to adults, childhood asthma risk correlates with the composition of the respiratory microbiome during early developmental stages. A prospective cohort study demonstrated that elevated relative abundances of *Veillonella* and *Prevotella* in the neonatal respiratory microbiome at 1 month of age are linked to an increased risk of asthma by 6 years [42], increased nasal colonization by *Haemophilus* and sustained low *Moraxella* abundance in infancy (0–12 months) are correlated with asthma diagnosis by 7 years of age [47], oropharyngeal *Neisseria* abundance during the second year of life shows a positive correlation with asthma incidence [48]. Elevated airway bacterial loads in asthmatic children are characterized by suppressed TNF-α/IL-1β production and upregulated CCL2/CCL17 expression [42]. Given that CCL2 and CCL17 serve as independent asthma biomarkers [49], these findings imply microbial-driven immune dysregulation may contribute significantly to asthmatic mechanisms.

### Lung cancer

Emerging research highlights the association between lung cancer and chronic inflammatory respiratory conditions, with mounting evidence underscoring the pathological interplay between lung cancer and COPD [50]. Prospective cohort analysis revealed a significant association between pulmonary *Veillonella* enrichment and adverse clinical outcomes in lung cancer patients [51], highlighting the potential involvement of lung microbiome dysbiosis in tumorigenesis and disease progression.

Patients with lung cancer exhibit elevated abundances of *Streptococcus* and *Prevotella* in the lower respiratory tract relative to healthy individuals [52, 53]. Compared to healthy subjects, salivary microbiome profiling of NSCLC patients revealed increased *Veillonella* colonization and decreased *Neisseria* abundance [54]. Research indicates that elevated abundances of *Streptococcus* and *Veillonella* in cancer patient bronchoalveolar lavage fluid (BALF) correlate with enhanced infiltration of inflammatory cells and activation of the PI3K/ERK signaling axis in bronchial epithelial cells [55]. Another study found that *Bacteroidota* enrichment in the lower respiratory tract exerts protective effects against lung carcinogenesis [56], compositional shifts in *Prevotella*, *Streptococcus*, and *Clostridium* within the lower airways show significant correlations with the magnitude of localized pulmonary inflammation [57].

### Interstitial lung disease

Compared with the healthy control group, patients with interstitial pneumonia exhibited a decreased abundance of *Streptococcus* in BALF, while the abundance of *Prevotella* and *Veillonella* increased [58]. Research has shown that the bacterial burden in BALF from patients with idiopathic pulmonary fibrosis (IPF) exceeds that of healthy controls, with microbial communities like *Streptococcus* and *Staphylococcus* linked to accelerated disease progression [59–62]. Consequently, factors affecting the respiratory microbial ecosystem could exert an indirect influence on the development and clinical outcomes of these diseases.

### Acute lung injury/acute respiratory distress syndrome (ALI/ARDS)

By constructing a standard ALI murine model through intratracheal LPS administration, researchers observed that *Firmicutes*, *Proteobacteria*, and *Bacteroidetes* in ALI mouse BALF exhibited positive correlations with total cell counts, while *Actinobacteria* was inversely associated [63]. Accumulating clinical evidence demonstrates marked dysbiosis in the lung microbiota of ALI/ARDS patients [64, 65], which may be mechanistically linked to injury-driven upregulation of inflammation-promoting factors that enhance bacterial proliferation and destabilize the pulmonary microenvironment.

Experimental studies revealed that hyperoxic exposure (FiO2 95%) induced lung microbiota alterations in mice as early as 24 h, preceding the development of hyperoxia-mediated lung injury observed at 72 h post-exposure [66]. This temporal pattern implies potential involvement of airway microbiota in the initiation of ALI/ARDS pathology.

Consequently, factors affecting the respiratory microbial ecosystem could exert an indirect influence on the development and clinical outcomes of these diseases.

## The impact of environmental factors on the respiratory microbiome

### Environmental temperature and humidity

Variations in ambient temperature can influence the composition of the respiratory microbiome. Wang et al. observed that periodic exposure to elevated environmental temperatures significantly altered the diversity of the respiratory microbiota in 11-week-old healthy Isa brown layer-type pullets. Specifically, the group exposed to 30 °C exhibited a marked increase in *Proteobacteria* and a concurrent decrease in *Firmicutes* in BALF compared to the control group maintained at 24 °C [67]. In female mice, concurrent exposure to 1 ppm ozone and a 36 °C thermal environment resulted in a reduction of *Firmicutes* in BALF, alongside an increase in total protein, tumor necrosis factor-alpha (TNF-α), and 8-hydroxydeoxyguanosine (8-OHdG) levels [68]. High-temperature environments can enhance the proliferation of pathogenic bacteria, including *Staphylococcus* aureus and *Streptococcus* pneumoniae, thereby diminishing the diversity of the normal respiratory microbiota. Conversely, low-temperature environments also impact the respiratory microbiota [69]. Clinical studies have indicated that prolonged inhalation of cold air at 26 °C can decrease the abundance of *Moraxella* species in the pharynx of healthy individuals [70]. Furthermore, rabbits exposed to 4 °C and 20 °C environments demonstrated significant differences in their lung microbiome composition, with a higher prevalence of *Proteus* at 4 °C compared to 20 °C [69].

The detrimental health implications of prolonged exposure to high temperatures are widely recognized. Additionally, unsuitable environmental humidity levels can adversely impact health, especially when compounded by high temperatures [71]. Studies have demonstrated that under hot and humid conditions, characterized by 31–33 °C with 91–95% relative humidity, the diversity of the gut microbiota in mice is observed to be distinct from that under standard conditions [72]. Within these environments, there is a notable reduction in *Firmicutes* and a corresponding increase in *Bacteroidota* in mouse feces [73]. Research investigating changes in oropharyngeal microbiota and their association with environmental conditions among school-aged children in Ivorian demonstrated an inverse relationship between *Neisseria* abundance and humidity levels [74].

### Air pollutants

Environmental pollution is emerging as a critical social issue in contemporary society. Sources such as industrial emissions, vehicular exhaust, combustion of fossil fuels, construction dust, and natural phenomena (e.g., sandstorms) contribute to the production of various air pollutants, including particulate matter (PM2.5, PM10), sulfur dioxide, carbon monoxide, nitrogen oxides, volatile organic compounds (VOCs), and ozone [75]. Research indicates that exposure to elevated concentrations of PM2.5 significantly impacts the lung microbiota in mice, leading to an increased abundance of *Bacteroidota* and *Firmicutes* [76]. Prolonged exposure to air pollutants can facilitate the colonization of pathogenic bacteria in the respiratory tract, such as *Streptococcus* pneumoniae, *Haemophilus* influenzae, and *Moraxella* [77]. A study examining the correlation between air pollutants during winter heating periods in northeastern China and respiratory microbiota imbalance revealed that individuals in highly polluted areas exhibited higher relative abundances of *Firmicutes* and *Proteobacteria* in their throat swabs compared to those in less polluted regions. Furthermore, as air pollution levels rise, the relative abundance of Fusobacterium and *Prevotella* species declines [78].

### Compound factors

Relative to environmental temperature, seasonal variation represents a more intricate factor, encompassing variations in temperature, precipitation, daylight duration, biodiversity, and human activities. The upper respiratory tract microbiota composition fluctuates across different seasons [79]. Research has indicated that during the fall and winter seasons, the nasopharyngeal abundance of *Proteobacteria* is elevated in children, whereas *Bacteroidota* becomes predominant in the spring [80].

The impact of altitude on the human body is also multifaceted. Regions at high altitude are defined by reduced atmospheric pressure, diminished oxygen partial pressure, intense ultraviolet radiation, low temperatures, arid conditions, and powerful winds [81]. The respiratory microbiota under normal circumstances encompasses both aerobic and anaerobic bacteria, which exhibit distinct adaptive mechanisms in response to fluctuating oxygen levels. In hypoxic conditions prevalent at high altitudes, there may be a reduction in the diversity of aerobic bacteria within the respiratory tract [82]. Nonetheless, scientific inquiry into this domain is exceedingly constrained.

## Mechanisms of environmental factors affect the respiratory microbiome

### Direct effects

Changes in environmental factors can directly influence the proliferation and reproduction of respiratory microorganisms, consequently impacting their prevalence in the respiratory tract. In vitro research has demonstrated that modifications to the culture environment can alter the quantity and vitality of respiratory microorganisms. One study explored the effects of environmental oxygen concentration (anaerobic environment, or aerobic environment provided by catalase) and temperature (30–39 °C) on the growth of multiple *Streptococcus* pneumoniae strains in vitro. Under aerobic conditions provided by catalase and at temperatures of 30–35 °C (normal nasal cavity temperature), the strains reached maximum density after 24 h of incubation. As the incubation temperature rose, the density of the strains diminished, with the minimum density recorded at temperatures between 37 °C and 39 °C. The majority of strains displayed optimal growth rates at temperatures of 35 °C to 37 °C and exhibited more rapid growth under aerobic conditions compared to anaerobic ones [83]. An additional study determined that the storage temperature of sputum samples significantly influenced the viability of *Streptococcus* pneumoniae, with a marked decrease in viability observed at 4 °C relative to room temperature [84].

Changes in environmental factors can also affect the virulence of respiratory microorganisms elicit inflammatory responses within the respiratory tract, thereby disrupting the respiratory microenvironment and affecting its stability. Under environmental conditions of 26 °C, the expression of the outer membrane protein UspA1 in *Moraxella* catarrhalis escalates, which facilitates bacterial adhesion to upper respiratory tract cells, augmenting its virulence and perturbing the normal respiratory microenvironment [85]. At 26 °C, *Moraxella* also enhances the bacteria-induced release of IL-8, exacerbating the inflammatory response [70].

### The gut-lung axis

The gut–lung axis has emerged as a prevalent area of investigation, delineating the intricate interactions between the gut microbiota and pulmonary health, and its pivotal role in modulating immune and inflammatory responses [86]. The BALF of ARDS patients exhibits a high prevalence and abundance of the gut-specific genus *Bacteroides*, which is linked to the severity of systemic inflammation [65]. Notably, *Bacteroides fragilis* has been demonstrated to contribute to the prevention of respiratory infections [87, 88]. Evidence has established an association between intestinal microbial changes and the development of asthma [89]. Emerging research has uncovered that the intestinal protozoan *Tritrichomonas musculis* regulates the lung immune environment, thereby mediating immune reactions associated with allergic airway inflammation and pulmonary infections [90].

Current researches indicate that short-chain fatty acids, produced by the gut microbiota, along with their extracellular vesicles, can permeate the pulmonary system via the circulatory or lymphatic pathways, thereby influencing the pulmonary microenvironment [91, 92]. Moreover, when the intestinal mucosal barrier is disrupted, gut microbiota have the potential to infiltrate the bloodstream or lymphatic system, subsequently reaching the lungs and modulating the composition of the pulmonary microbiota [92]. Persistent exposure to elevated temperatures or intense physical exertion under such conditions can amplify heat stress, intensify oxidative stress within the intestines, impair intestinal epithelial cells and tight junction proteins, and erode the intestinal physical barrier. Consequently, this enables gut bacteria to invade the bloodstream and migrate to the lungs, subsequently impacting the lung microbiome [93]. Nonetheless, comprehensive research focusing on the precise alterations within the gut–lung axis under various environmental conditions and their association with the respiratory microbiome and pulmonary diseases remains sparse.

## Conclusion and outlook

As global warming intensifies and air pollution worsens, the impact of environmental factors on health is gaining increasing attention. The respiratory tract is directly exposed to atmospheric physical and chemical parameters, making it a keyway through which environmental factors affect human health. Existing evidence has indicated that environmental temperature, humidity, and air pollutants potentially alter the incidence of respiratory diseases by affecting the respiratory microbiome. However, there is a dearth of comprehensive and systematic research in this area. Additionally, investigations into microorganisms aid in preventing both the initiation and advancement of diseases. Existing evidence confirms that probiotic intake reduces susceptibility to respiratory infections [94]. Future studies should aim to investigate the variations in the respiratory microbiome under diverse environmental conditions, to enhance our understanding of its role in perpetuating homeostasis and to clarify its regulatory mechanisms. Such investigations could yield novel insights and therapeutic strategies for disease prevention and management.

## Data Availability

No datasets were generated or analysed during the current study.
